# Evaluation of the Distortion of Essix Retainer Formed From Biostar Machine Using Intraoral Digital Scans: An In Vitro Study

**DOI:** 10.7759/cureus.66858

**Published:** 2024-08-14

**Authors:** Mahima Sehgal, Usha Shenoy, Ananya Hazare, Himija Karia, Pritam R Khorgade, Nivedita Nandeshwar, Sangeeta Sangeetabhattacharya

**Affiliations:** 1 Department of Orthodontics and Dentofacial Orthopedics, Ranjeet Deshmukh Dental College & Research Centre, Nagpur, IND

**Keywords:** retainers, relapse, retention, biostar machine, vaccum formed retainer, essix retainer

## Abstract

Introduction

Retention is essential to prevent unwanted tooth movement due to growth changes, to allow the gingival and periodontal tissues affected by orthodontic treatment to realign, and to stabilize teeth that have been moved to potentially unstable positions, thus reducing the risk of relapse. This study aimed to evaluate the distortion of Essix retainers over time to enhance their retention and stability.

Methods

Patients who visited the Department of Orthodontics and Dentofacial Orthopedics at Ranjeet Deshmukh Dental College & Research Centre, Nagpur, India, after completing their orthodontic treatment were included in the study, according to the established inclusion and exclusion criteria. A total of 26 patients participated. Each patient received an Essix retainer fabricated from a Duran+ Biostar round sheet (1 mm thickness) using a Biostar machine based on their post-debonded maxillary cast. The patients were instructed on the correct insertion and removal of the Essix retainer. The inner surface of the retainers was scanned at one month, three months, and six months using an intraoral digital scanner. These scans were analyzed and compared for distortion at different time intervals using Medit software.

Results

The Essix retainers exhibited varying degrees of distortion at different time intervals. Notably, distortion was more significant in the posterior region compared to the anterior region. Additionally, distortion increased over time, with the least amount observed at one month and progressively worsening by the sixth month. Specifically, the average distortion in the posterior region ranged from 0.133 mm after the first month to 0.304 mm after six months. In contrast, the average distortion in the anterior region was lower, ranging from 0.057 mm at one month to 0.068 mm at six months.

Conclusions

Distortion was more pronounced on the posterior surface of the Essix retainer compared to the anterior region.

## Introduction

“It’s not over until it’s over” is a saying that applies perfectly to orthodontic treatments [1]. Maintaining the teeth in their proper positions is a difficult task that does not end with a successful orthodontic treatment. Both the patient and the orthodontist bear responsibility for this. Creating an optimal occlusion that is morphologically stable, aesthetically pleasing, and functional is the aim of orthodontic therapy [1]. The outcomes attained at the conclusion of active treatment are not always stable over the long run, even with a correct diagnosis and meticulously executed treatment mechanics [2].

The most difficult and uncertain phase of orthodontic treatment is retention, and it is still debatable which type of retention is clinically the most beneficial [3]. Retention is required to prevent unwanted tooth movement resulting from changes in growth, to allow the gingival and periodontal tissues impacted by orthodontic tooth movement to realign, and to stop teeth that have been moved to an inherently unstable position from having a predisposition to relapse [2]. Relapse, or the propensity for the initial malocclusion to recur, is a consequence of skipping the retention phase. Relapse may result from imbalances in the soft tissues of the face, periodontal tissues, gingival fibers, or occlusion. Furthermore, in growing patients, negative growth trends can lead to unfavorable changes following the removal of the orthodontic fixed appliance at the conclusion of active treatment [2].

Following active orthodontic procedures, a variety of removable retainers have been utilized to keep teeth in their final, aesthetically pleasing, and functional positions. The two removable retainers that are most frequently prescribed in orthodontic practices are Hawley retainers (HRs) and vacuum-formed retainers (VFRs) [3,4]. It is crucial that the orthodontist select a removable retainer type that offers high levels of acceptance and strong retention, which may improve the long-term stability of the outcomes [3].

In 1919, Charles Hawley first utilized the HR, and it has since become extensively used. It consists of Stahl or Adams clasps, an acrylic mass that contacts the palatal mucosa and the surfaces of each tooth in the dental arch, and a stainless-steel wire labial bow. Controlling the incisor torque and permitting posterior teeth to move vertically are two benefits of the labial bow, which originates from four or six anterior teeth. The foremost shortcomings of the HR are associated with its extensive coverage of the palate and the visibility of the labial bow [2]. Extensive palatal coverage can be uncomfortable in the beginning, interfere with normal speech patterns, and, with prolonged use, can cause fit issues.

In 1971, Ponitz described a clear thermoplastic retainer as an alternative to the conventional removable retainer. This kind of retainer is roughly one-third less expensive than a traditional HR and is strong, aesthetically pleasing, and simple to clean. The literature referred to them as Essix retainers, VFRs, or clear overlay retainers [3]. These thermoplastic retainers, which are removable, are composed of polyethylene and polypropylene polymers. Polyethylene polymers have the advantage of being more translucent, aesthetically pleasing, and bondable to acrylic. The VFRs are made using a vacuum machine that draws the thermoplastic material onto the working cast while adapting a heat-softened plastic under negative pressure. Materials like Duran, Essix ACE, C+, and Tru-Train, which are readily available on the market, are used to make these retainers. Other brands that supply them to the market include Vivera, Essix, and Zendura. Thermoplastic sheets of 0.75, 1, 1.5, and 2 mm thickness are available for them. The thickness of VFRs has not yet been subject to a unified standard [2,3].

Thermoforming is a process used in manufacturing when plastic is heated to a temperature that allows it to be formed into the desired shape. The two main thermoforming techniques used in orthodontics to make retainers are vacuum form and pressure form. The vacuum form method conforms to heat-softened plastic by applying negative pressure to a cast using a vacuum machine. In the pressure form method, heat is utilized to soften the plastic, which is then pushed by a mixture of positive and negative air pressure to cover the cast and form the thermoform retainer [5].

Two different kinds of plastic thermoforming machines can be used to produce VFRs: pressure machines, which are considered superior as they use positive pressure to force heat-softened plastic over the plaster mold at the end of treatment (e.g., Biostar™) and vacuum machines, which form the heat-softened plastic to the mold using negative pressure. These VFRs have the advantages of excellent aesthetics, affordability, and simplicity of construction. The most frequent drawbacks are limited vertical settling of teeth, occlusal wear, and fractures (poor wear resistance and durability). VFRs are typically 0.75 mm or 1 mm thick. Numerous studies on the subject of periodontal health, patient comfort (including speech, mastication, accommodation duration, compliance, and patient satisfaction), retainer effectiveness, drawbacks, and expenses have been published in the past several decades [2-4,6].

Appliances made of thermoformed plastic are lacking in two areas: fit and resistance to wear. A precise fit is essential in every thermoformed application, from the time the appliance is first placed until it is used. If the retentive gingival undercuts are not well defined, the device will fit poorly; if the undercuts are severe, the appliance will be too tight. The degree to which the undercuts conform to the contact points determines the fit and adaptation. An incorrect adaptation could be a sign of demineralization of the enamel and a potential shift in bite [7].

In order to create an orthodontic retainer that offers superior retention and stability before the retainer begins to distort, the purpose of our study was to assess the distortion of Essix retainers made from Biostar sheets using intraoral digital scanners after specific time intervals.

## Materials and methods

This study aimed to assess the distortion of Essix retainers made from Biostar sheets using intraoral digital scanners at specific time intervals. Approval was obtained from the institutional ethical committee (approval number IEC/VSPMDCRC/20/2022). Patients who visited the Department of Orthodontics and Dentofacial Orthopedics after completing their orthodontic treatment were included based on established inclusion and exclusion criteria. Using G*Power version 3.0.1 (Franz Faul, Universität Kiel, Germany), a sample size of 26 individuals was calculated to achieve 80% power to detect significant differences, with an effect size of 0.5 and a significance level of 0.05.

Appropriate study instruments

The armamentarium used to make Essix retainers are a Duran+ Essix thermoforming hard sheet (around 1 mm thick), a Biostar machine, and a dental impression cast. The armamentarium used for the scanning process includes CAD/CAM spray, an intraoral digital scanner, an Essix retainer, and a maxillary impression cast. The armamentarium used to evaluate distortion in the Essix retainer includes Medit Software, a digital scan of the post-treatment cast of the patient, and a digital scan of the inner surface of the Essix retainer.

Inclusion and exclusion criteria

The patients who had visited the orthodontics department completed the active phase of their treatment (1.5-2 years) and were in the retention phase; specifically, those who had been provided with an Essix retainer for this phase were selected for the study. Patients with a history of bruxism, nail biting, lip biting, poor periodontal condition, and smoking were excluded from the study.

Method

**Figure 1:**
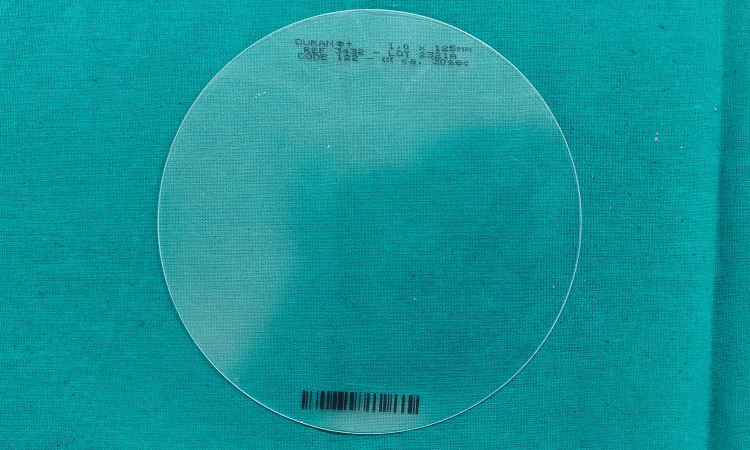
Duran+ Biostar round sheet

**Figure 2:**
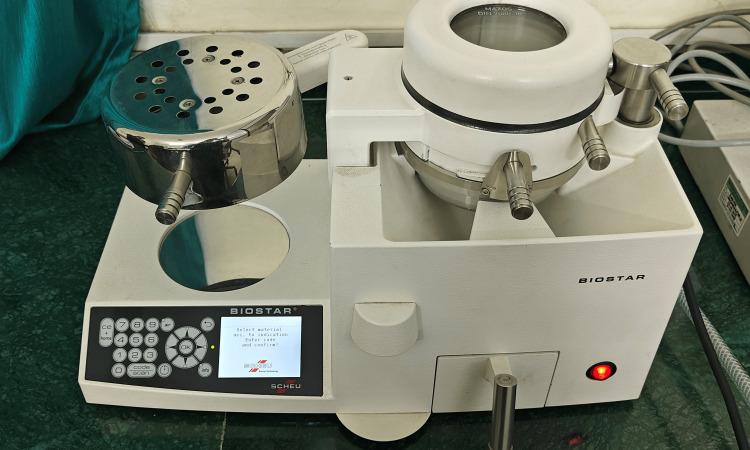
Biostar machine

Based on the inclusion and exclusion criteria, 26 patients who had finished their orthodontic treatment and were in the retention phase were chosen for this study. Alginate impressions were taken immediately after debonding of the fixed appliance, and the cast was formed using dental stone. An Essix retainer using a Duran+ Biostar round sheet (1 mm thickness) (Figure 1) was fabricated on a Biostar machine (Figure 2) using the post-debonded cast of the patient.

**Figure 3:**
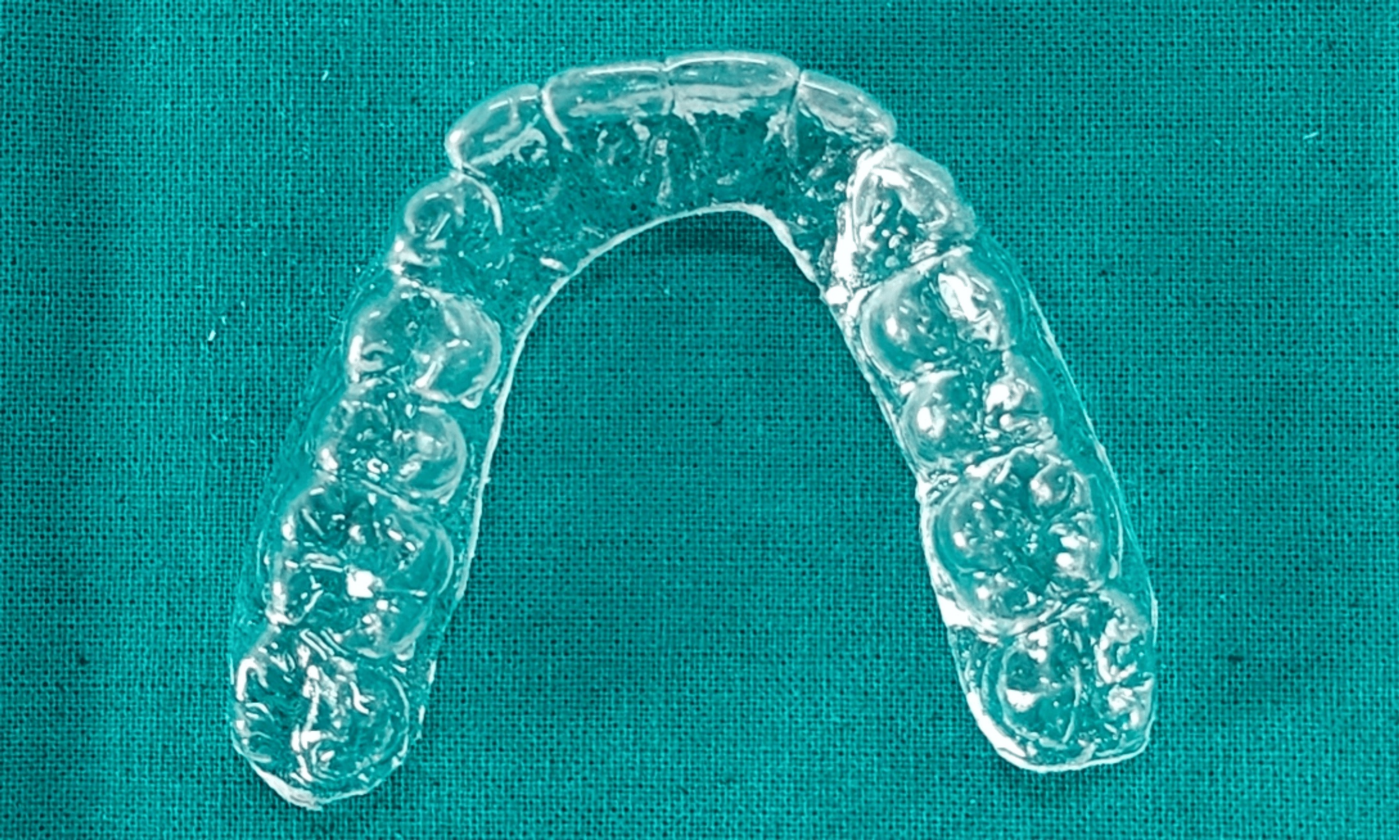
Essix retainer after trimming

The maxillary cast is placed in the model cup filled with stainless steel pellets. The Biostar sheet is scanned by the Biostar machine. The Biostar sheet (1 mm) then undergoes the heating process for 30 seconds. After the heating process, the heated Biostar sheet is adapted to the maxillary cast, and it undergoes a cooling process. The Essix retainer is retrieved from the Biostar machine, trimmed, and delivered to the patient (Figure 3). The patient was given instructions on the proper insertion and removal of the Essix retainer. The patient was instructed to remove the retainer while eating and drinking beverages.

**Figure 4:**
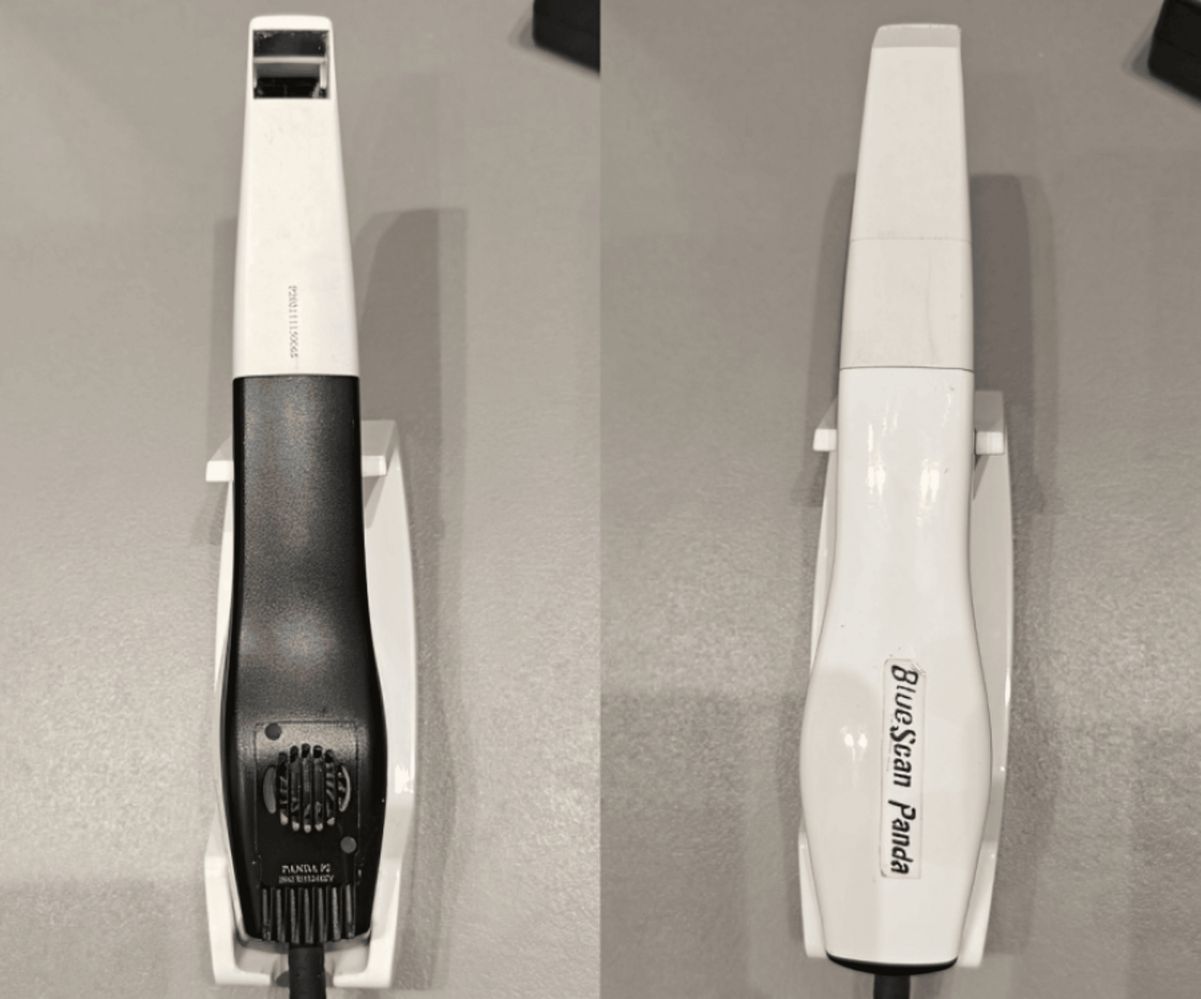
Intraoral digital scanner

**Figure 5:**
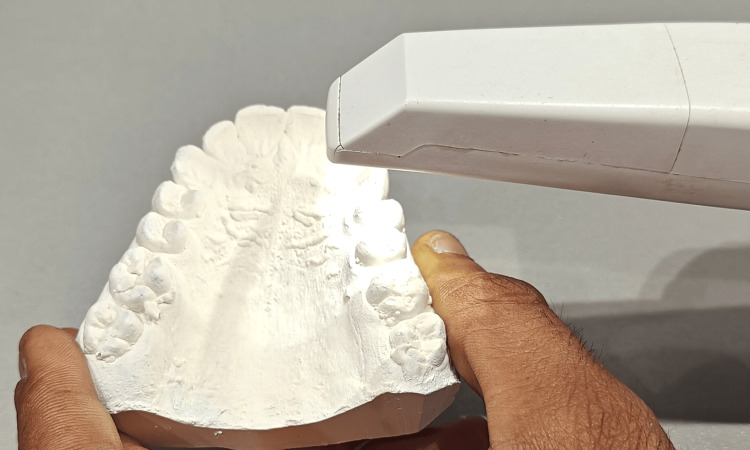
Maxillary cast scanning using an intraoral digital scanner

**Figure 6:**
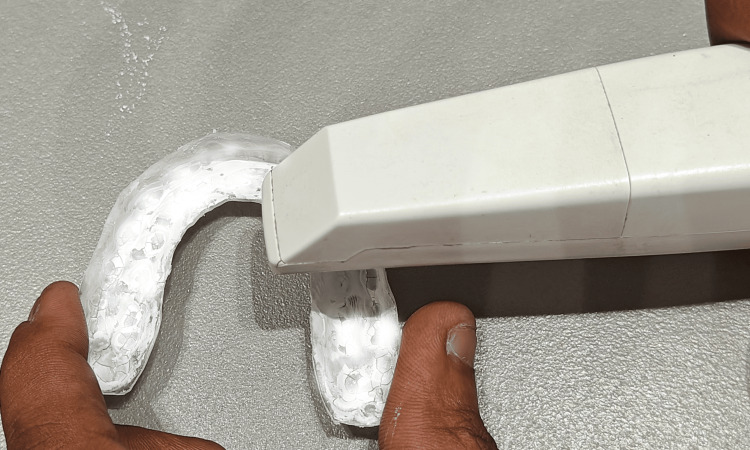
Scanning of the inner surface of the Essix retainer using an intraoral digital scanner

An intraoral digital scanner (Figure 4) was used to scan the cast and the Essix retainer at different time intervals. The occlusal surface of the maxillary post-orthodontic treatment cast was scanned using an intraoral digital scanner (Figure 5). The scanning process was done by a single trained technician. The inner surface of the Essix retainer was blocked out and made opaque by using a CAD/CAM spray. After a duration of one month, the inner surface of the retainer was also scanned using the same intraoral digital scanner (Figure 6). Similar scans of the retainer were repeated after a duration of three months and six months.

**Figure 7:**
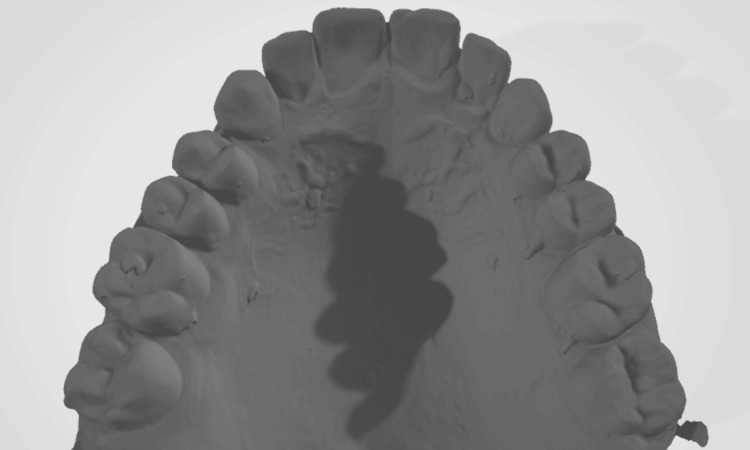
Digital STL file of the post-orthodontic cast scan STL, stereolithography

**Figure 8:**
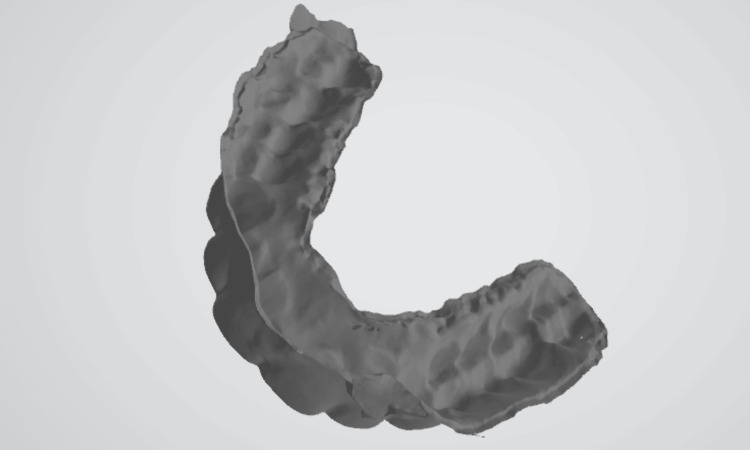
Digital STL file of the inner surface of the retainer STL, stereolithography

A digital stereolithography (STL) file of the post-orthodontic cast scan (Figure 7) and a digital STL file of the inner surface of the retainer at different time intervals were generated. The STL files of the post-orthodontic cast and the inner surface of the retainer (Figure 8) were analyzed using Medit software.

**Figure 9:**
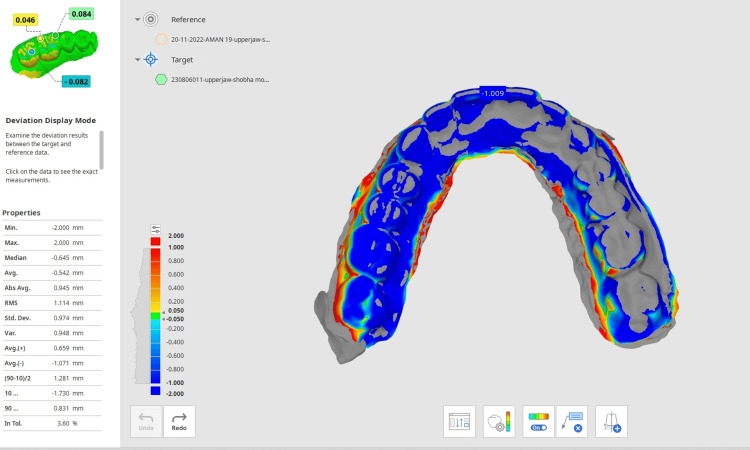
Superimposition of the maxillary cast STL file and retainer STL file in Meddit software STL, stereolithography

**Figure 10:**
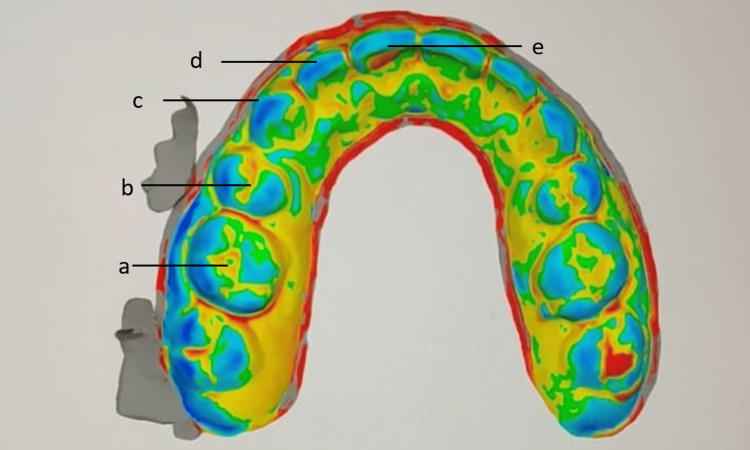
Preselected points to measure the deviation between the maxillary cast and the Essix retainer (a) Central fossa of molars. (b) Central groove of premolars. (c) Cusp tip of canine. (d) Mid-incisal edge of the lateral incisor. (e) Mid-incisal edge of the central incisor.

The digital scan of the inner surface of the retainer taken at different time intervals was superimposed on the digital scan of the post-orthodontic cast individually in the Medit software to evaluate the distortion in the retainer (Figure 9). The distortion was evaluated at preselected points on each tooth of the maxillary arch. The preselected points were the central fossa of the molars, the central groove of the premolars, the cusp tip of the canine, the mid-incisal edge of the lateral, and the central incisors (Figure 10). The deviation was observed in the retainer at different time intervals and plotted on the graph.

## Results

The purpose of this study is to evaluate the distortion of Essix retainers after a certain time period so as to provide better retention and stability. The null hypothesis of the study was that there was no significant distortion in the Essix retainer at different time intervals formed from the Biostar machine.

Table 1 presents the comparison of the change in distortion of the retainer in individual teeth over different time intervals using the ANOVA test and Friedman test (p ≤ 0.05). There was a significant difference in the one-month, three-month, and six-month distortion values of each tooth, with values showing an increase from a one-month interval to a six-month interval.

**Table 1:** Comparison of the change in distortion of the retainer in individual teeth over different time intervals Repeated measures ANOVA test; ^#^ Friedman test; * indicates a significant difference at p ≤ 0.05 R1, retention after one month; R2, retention after three months; R3, retention after six months

Tooth	R1		R3		R6		p-value
	Mean	SD	Mean	SD	Mean	SD	
16	0.133	0.011	0.218	0.006	0.304	0.011	<0.001*
15	0.116	0.011	0.15	0.012	0.181	0.021	<0.001*
14	0.118	0.008	0.15	0.006	0.179	0.015	<0.001*
13	0.047	0.007	0.055	0.005	0.062	0.006	<0.001*
12	0.061	0.007	0.065	0.007	0.069	0.008	<0.001*
11	0.062	0.008	0.066	0.008	0.07	0.009	<0.001*
21	0.062	0.007	0.065	0.007	0.069	0.007	<0.001*
22	0.06	0.006	0.064	0.006	0.068	0.007	<0.001*
23^#^	0.051	0.009	0.061	0.012	0.071	0.022	<0.001*
24^#^	0.123	0.014	0.145	0.01	0.168	0.015	<0.001*
25	0.119	0.014	0.143	0.01	0.167	0.016	<0.001*
26	0.134	0.012	0.22	0.006	0.304	0.009	<0.001*

Table 2 presents the pairwise comparison of the change in distortion of the retainer in individual teeth over different time intervals using the Bonferroni test and the post hoc Wilcoxon signed rank test (p ≤ 0.05). In each tooth, there was a significant difference between one-month and three-month distortion values (one month < three months). Also, the difference between one-month and six-month distortion values was statistically significant for each tooth (one month < six months). When the change in distortion values from three months to six months was compared, the change was statistically significant (three months < six months).

**Table 2:** Pairwise comparison of the change in distortion of the retainer in individual teeth over different time intervals Adjustment for multiple comparisons: Bonferroni test; ^#^ Post hoc Wilcoxon signed rank test; * indicates a significant difference at p ≤ 0.05 R1, retention after one month; R2, retention after three months; R3, retention after six months

Tooth	R1 vs. R3		R1 vs. R6		R3 vs. R6	
	Difference	p-value	Difference	p-value	Difference	p-value
16	-0.086	<0.001*	-0.171	<0.001*	-0.086	<0.001*
15	-0.034	<0.001*	-0.064	<0.001*	-0.03	<0.001*
14	-0.032	<0.001*	-0.06	<0.001*	-0.028	<0.001*
13	-0.008	<0.001*	-0.015	<0.001*	-0.007	<0.001*
12	-0.004	<0.001*	-0.008	<0.001*	-0.004	<0.001*
11	-0.004	<0.001*	-0.007	<0.001*	-0.004	<0.001*
21	-0.003	<0.001*	-0.007	<0.001*	-0.003	<0.001*
22	-0.004	<0.001*	-0.008	<0.001*	-0.004	<0.001*
23^#^	-0.01	<0.001*	-0.02	<0.001*	-0.01	<0.001*
24^#^	-0.023	<0.001*	-0.046	<0.001*	-0.023	<0.001*
25	-0.025	<0.001*	-0.049	<0.001*	-0.024	<0.001*
26	-0.085	<0.001*	-0.169	<0.001*	-0.084	<0.001*

Table 3 presents the comparison of the change in distortion of the retainer among anterior teeth over different time intervals using the Friedman test and post hoc Wilcoxon signed rank test at p ≤ 0.05. From one month to six months, there was an increase in the distortion of the retainers placed over the anterior teeth, and the change was statistically significant. A pairwise comparison showed that the difference between one-month and three-month distortion values of anterior teeth was significant (one month < three months). Also, the difference between one-month and six-month distortion values was statistically significant for the retainers placed over the anterior teeth (one month < six months). When the change in distortion values from three months to six months was compared, the change was statistically significant (three months < six months).

**Table 3:** Comparison of changes in distortion of the retainer among anterior teeth over different time intervals Friedman test; post hoc Wilcoxon signed rank test; * indicates a significant difference at p ≤ 0.05 R1, retention after one month; R2, retention after three months; R3, retention after six months

Interval	Mean	SD	p-value	Pairwise comparisons
R1	0.057	0.006	<0.001*	R1 vs. R3: <0.001*
R3	0.063	0.006		R1 vs. R6: <0.001*
R6	0.068	0.008		R3 vs. R6: <0.001*

Table 4 presents the comparison of the change in distortion of the retainer among right posterior teeth over different time intervals using the Friedman test and the post hoc Wilcoxon signed rank test (p ≤ 0.05). From one month to six months, there was an increase in the distortion of the retainers placed over the right posterior teeth, and the change was statistically significant. A pairwise comparison showed that the difference between one-month and three-month distortion values of the right posterior teeth was significant (one month < three months). Also, the difference between one-month and six-month distortion values was statistically significant for the retainers placed over the right posterior teeth (one month < six months). When the change in distortion values from three months to six months was compared, the change was statistically significant (three months < six months).

**Table 4:** Comparison of changes in distortion of the retainer among right posterior teeth over different time intervals Friedman test; post hoc Wilcoxon signed rank test; * indicates a significant difference at p ≤ 0.05 R1, retention after one month; R2, retention after three months; R3, retention after six months

Interval	Mean	SD	p-value	Pairwise comparisons
R1	0.122	0.01	<0.001*	R1 vs. R3: <0.001*
R3	0.174	0.007		R1 vs. R6: <0.001*
R6	0.224	0.015		R3 vs. R6: <0.001*

Table 5 presents the comparison of the change in distortion of the retainer among left posterior teeth over different time intervals using the ANOVA test and Bonferroni test (p ≤ 0.05). From one month to six months, there was an increase in the distortion of the retainers placed over the left posterior teeth, and the change was statistically significant. A pairwise comparison showed that the difference between one-month and three-month distortion values of left posterior teeth was significant (one month < three months). Also, the difference between one-month and six-month distortion values was statistically significant for the retainers placed over the left posterior teeth (one month < six months). When the change in distortion values from three months to six months was compared, the change was statistically significant (three months < six months).

**Table 5:** Comparison of changes in distortion of the retainer among left posterior teeth over different time intervals Repeated measures ANOVA test; adjustment for multiple comparisons: Bonferroni test; * indicates a significant difference at p ≤ 0.05 R1, retention after one month; R2, retention after three months; R3, retention after six months

Interval	Mean	SD	p-value	Pairwise comparisons
R1	0.125	0.013	<0.001*	R1 vs. R3: <0.001*
R3	0.171	0.007		R1 vs. R6: <0.001*
R6	0.216	0.013		R3 vs. R6: <0.001*

Table 6 presents the comparison of distortion of the retainer between right and left posterior teeth over different time intervals using the independent t-test and Mann-Whitney test (p ≤ 0.05). After one month, there was no difference in the distortion between the right and left posterior teeth. After three months, there was no difference in the distortion between the right and left posterior teeth. However, after six months, there was a significant difference in the distortion between the right and left posterior teeth, with retainers placed over the right posterior teeth showing significantly greater distortion as compared to the retainers placed over the left posterior teeth.

**Table 6:** Comparison of distortion of the retainer between right posterior teeth and left posterior teeth over different time intervals ^$^ Independent t-test; ^#^ Mann-Whitney test; * indicates a significant difference at p ≤ 0.05 R1, retention after one month; R2, retention after three months; R3, retention after six months

Interval	Right posterior		Left posterior		Difference	p-value
	Mean	SD	Mean	SD		
R1^#^	0.122	0.01	0.125	0.013	-0.003	0.287
R3^$^	0.174	0.007	0.171	0.007	0.003	0.079
R6^$^	0.224	0.015	0.216	0.013	0.008	0.042*

Thus, the null hypothesis was rejected due to the statistically significant difference in distortion of the Essix retainer at different time intervals formed from the Biostar machine.

## Discussion

“Retention is one of the most difficult problems in orthodontia; in fact, it is the problem,” as Oppenheim noted in 1934. This statement remains pertinent even 75 years later [8]. Moyers defined retention as “the process of keeping teeth in the treated position for the necessary duration to maintain the results following orthodontic treatment.” To preserve arch shape and minimize the risk of relapse, various retention devices have been employed after orthodontic treatment [9]. The primary goal of retention is “to keep the teeth in their corrected positions at the end of active orthodontic treatment” [2]. Introduced by Sheridan et al. in 1993, the Essix retainer - a vacuum-formed, removable retainer - has gained widespread use in recent years. These retainers, made from polyvinyl siloxane sheets, cover every surface of the teeth [2].

In spite of the fact that Essix retainers gained popularity and are preferred more by patients because of their esthetics and comfort, these retainers have a few drawbacks. The Essix retainers are more prone to fracture in the posterior region after their use for a certain time period; they tend to become loose by improper insertion and removal of the retainer, and these retainers also wear after a certain time duration [4,10].

Therefore, this study evaluated the distortion of Essix retainers formed from Biostar sheets using an intraoral digital scanner after certain time intervals for a duration of 6 months so as to provide an orthodontic retainer that provides better retention and stability. The distortion was more pronounced in the posterior region because there was more biting force in the posterior region as compared to the anterior region. Further, the distortion increased with the elapsed time, with the with the distortion being lowest after one month and gradually increasing till the sixth month. The average distortion in the posterior region ranged from 0.133 mm at the end of the first month to 0.304 mm at the end of six months. The average distortion in the anterior region was lower than the posterior region and ranged from 0.057 mm at the end of the first month to 0.068 mm at the end of six months.

It has already been observed in the literature that measurements up to 0.50 mm are typically regarded as clinically suitable for the assessment of a digital articulation. Every retainer used in this study produced measurements that were within 0.50 mm. Furthermore, no information is available regarding the maximum permissible distance that can be maintained between clear retainers and their master casts in order to assess the degree of distortion the retainer has experienced and to make clinical use of them accurately. The fit was assessed by measuring the distance between the retainer and its reference model at specified reference points. Nonetheless, this study revealed that the retainer’s distortion varied significantly depending on the time intervals examined. Its main objective was to determine how long the Essix retainer could remain functional before warping and causing problems during the retentive phase. It should nevertheless be mentioned that distortion does not determine how long VFRs last. Another reason a retainer might not work is if it breaks, gets misplaced, or stops fitting.

The findings of this investigation agree, at least in part, with those of Asma Ashari et al.’s study. Over the course of a 12-month retention period, they examined the therapeutic efficacy of modified VFRs (mVFRs) and HRs. They discovered that the mFVR group had more reported incidences of retainer fracture than the HR group. The thermoplastic material in mVFR has semi-elastic and resilient qualities that may have contributed to the breaking by making it more susceptible to parafunctional and functional activities [5]. Similar results were observed in a study conducted by Saleh et al., wherein after six months of use, the participants thought that the HRs were substantially more durable than the VFRs [11]. By comparing the fit of conventionally manufactured and 3D-printed transparent retainers to the original digital models, David J. Cloe evaluated their trueness. He concluded that conventional VFRs showed the least deviation from the original reference models. Demir et al. additionally looked at the clinical efficacy of Hawley and clear retainers at one and two years following the treatment period, and they found that clear retainers were superior for the retention of mandibular anterior teeth [9].

The findings of this study offer insights into the durability of thermoplastic retainer materials. The null hypothesis of this study is rejected, and deformation of the vacuum-formed Essix retainer was seen after six months of usage. In the results of this study, it is indicated that the vacuum-formed Essix retainer of the patient must be examined regularly at different time intervals so as to check for fractures or deformation in the Essix retainer and to provide better retention and stability after orthodontic treatment is complete.

However, there are certain limitations to this study. The precision of the intraoral digital scanner to record undercuts is of utmost importance. The intraoral digital scanner used in this study was unable to record the minute undercuts. Different levels of the masticatory force of individual patients affected the wear resistance of the vacuum-formed Essix retainer. Patients who did not follow the ideal path of insertion and removal of the Essix retainer showed significant deformation of the Essix retainer. The transparent vacuum-formed Essix retainer was blocked out using CAD/CAM spray to make it opaque so that the inner surface could be scanned easily. There was no standard method of application of a uniform layer of the CAD/CAM spray on the inner surface of the retainer, which led to uneven application and uneven thickness of the spray. While scanning the inner surface of the retainer, this uneven layer of CAD/CAM spray was seen as a deformation of the retainer.

## Conclusions

Distortion was more pronounced on the posterior surface of the Essix retainer compared to the anterior region. Over time, distortion increased, with the lowest distortion observed at one month and a gradual rise until the sixth month. In the posterior region, average distortion ranged from 0.133 mm at one month to 0.304 mm at six months. In contrast, the anterior region experienced less distortion, ranging from 0.057 mm at one month to 0.068 mm at six months.

It is well established that inconsistent use of a retainer can lead to poor fit, resulting in pain and discomfort. Several factors influence patient compliance, including age, gender, motivation, type of retainer, time since debonding, and understanding of proper retainer use. Effective prevention of relapse hinges on strong patient motivation and comfort with the retainer.
